# Oxidative Stress and Air Pollution: Its Impact on Chronic Respiratory Diseases

**DOI:** 10.3390/ijms24010853

**Published:** 2023-01-03

**Authors:** Martha Patricia Sierra-Vargas, Josaphat Miguel Montero-Vargas, Yazmín Debray-García, Juan Carlos Vizuet-de-Rueda, Alejandra Loaeza-Román, Luis M. Terán

**Affiliations:** 1 Departmento de Investigación en Toxicología y Medicina Ambiental, Instituto Nacional de Enfermedades Respiratorias Ismael Cosío Villegas (INER), Ciudad de México 14080, Mexico; 2 Departmento de Investigación en Inmunogenética y Alergia, Instituto Nacional de Enfermedades Respiratorias Ismael Cosío Villegas (INER), Ciudad de México 14080, Mexico

**Keywords:** oxidative stress, respiratory diseases, air pollution

## Abstract

Redox regulation participates in the control of various aspects of metabolism. Reactive oxygen and nitrogen species participate in many reactions under physiological conditions. When these species overcome the antioxidant defense system, a distressed status emerges, increasing biomolecular damage and leading to functional alterations. Air pollution is one of the exogenous sources of reactive oxygen and nitrogen species. Ambient airborne particulate matter (PM) is important because of its complex composition, which includes transition metals and organic compounds. Once in contact with the lungs’ epithelium, PM components initiate the synthesis of inflammatory mediators, macrophage activation, modulation of gene expression, and the activation of transcription factors, which are all related to the physiopathology of chronic respiratory diseases, including cancer. Even though the pathophysiological pathways that give rise to the development of distress and biological damage are not fully understood, scientific evidence indicates that redox-dependent signaling pathways are involved. This article presents an overview of the redox interaction of air pollution inside the human body and the courses related to chronic respiratory diseases.

## 1. Introduction

In the cell’s metabolic process, reactive oxygen and nitrogen species (ROS/RNS) are produced, which have high reactivity; they are free radicals. Free radicals come from redox reactions, radiolysis, photolysis, and hemolytic fission, where chemical bonds break and each newly created fragment preserves one of the bounded initial electrons [1]. Even though they are reactive molecules, they also contribute to different cellular processes, such as protein phosphorylation, secondary messengers, the activation of transcription factors, immune responses, and apoptosis. The cellular organelles that contribute to the endogen generation of ROS/RNS are the mitochondria, through the electron transport chain that produces the primary ROS, the superoxide anion radical (O_2_^•−^), which can further interact with other molecules to generate secondary ROS such as hydrogen peroxide (H_2_O_2_) and the hydroxyl radical (HO^•^). Peroxisomes produce H_2_O_2_ under physiological conditions, and nicotine adenine dinucleotide phosphate (NAD(P)H) oxidase in phagocytes generates O_2_^•−^ through respiratory bursts to destroy bacteria [2]. In parallel, RNS are also mainly produced under hypoxic conditions that activate the nitric oxide synthases in the mitochondria and phagocytic cells during respiratory bursts. Moreover, Toll-like receptors (TLR) such as TLR1, 2, and 4 can produce ROS by recruiting mitochondria to macrophage phagosomes [3]. 

**Figure 1:**
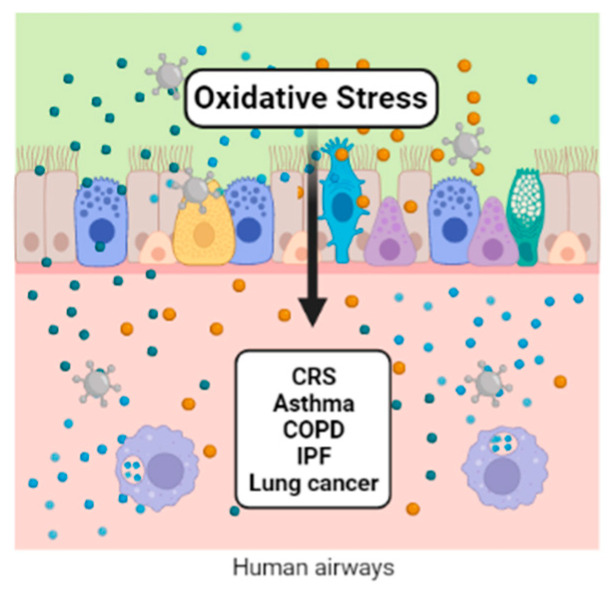
Oxidative stress activates the lungs’ epithelial cells, generating inflammatory mediators that participate in the macrophage activation and the modulation of gene expression and transcription factors. All of them are implicated in numerous respiratory diseases.

All those mechanisms are driven at the physiological level of ROS/RNS, known as oxidative eustress. Cellular antioxidant mechanisms maintain eustress, and when the formation of ROS/RNS overwhelms the cell’s antioxidant defense, molecular damage is produced, characterized as oxidative stress or distress [4]. Exogenous factors contributing to the generation of ROS/RNS include exposure to environmental pollutants, such as heavy metals (Cd, Hg, Pb, Fe, and As), certain drugs (cyclosporine, tacrolimus, gentamycin, and bleomycin), chemical solvents, cooking (smoked meat, used oil, and fat), cigarette smoke, vaping, alcohol, and radiation [5]. There is growing evidence that air pollution enhances oxidative stress and contributes to several diseases, from airway illnesses to DNA damage [6]. This review focuses on the participation of oxidative stress in the pathophysiology of respiratory tract diseases (Figure 1).

## 2. Chronic Rhinosinusitis (CRS) and Nasal Polyps (NP)

Chronic rhinosinusitis (CRS) is a chronic inflammation of the nose and paranasal sinuses, with a wide range of clinical phenotypes. This heterogeneous disease has an incidence of approximately 5%, significantly impacting the patients’ quality of life and productivity. One-third of the world’s population with CRS has nasal polyps (CRSwNP) [7]. A Type 2 inflammation mediated by the mast cells is present in CRS in response to increased oxidative stress. It has been suggested that air pollution causes an inflammatory change in the respiratory epithelium associated with CRS. However, there are few studies on the impact of air pollution and oxidative stress on the development of CRS. Recently, Patel and colleagues studied the relationship between levels of particulate air pollution (PM_2.5_) and the pathogenesis of CRS. They found that exposure to ambient air pollutants may contribute to the pathogenesis of this disease. Ozone is another air component linked to higher tissue inflammation, eosinophilic aggregates, and Charcot–Leyden crystals in CRSwNP patients evaluated in one study [8]. Another critical aspect investigated was whether socioeconomic status and exposure to airborne pollutants such as PM_2.5_, black carbon (BC), and NO_2_ increased the disease’s severity. The results showed that lower socioeconomic status predicted higher exposure to air pollution and increased disease severity in patients with CRS [9]. 

A study evaluated occupational airborne exposure and the severity of CRS [10]. The impact of exposure to vapors, gases, dust, fumes, fibers, and mist on 113 patients with CRSwNP, 96 with CRS without nasal polyps (CRSsNP), and 96 patients with aspirin-exacerbated respiratory disease (AERD) were evaluated. Patients exposed to these air contaminants required higher steroid doses than nonexposed patients. Contrary to other reports, this study found that PM_2.5_ and BC did not have a high impact on disease severity. On the other hand, Zheng and colleagues (2020) studied the role of nicotinamide adenine dinucleotide phosphate (NADPH) oxidase in CRSwNP. This oxidase has been associated with the pathogenesis of CRSwNP. Zheng et al. found, by Western blotting and real-time PCR, that this oxidase is increased in the nasal polyps of patients. These findings suggest that oxidative stress plays a role in the pathogenesis of CRSwNP [11]. The expression level of several oxidative stress and inflammation-related genes provided valuable information on the impact of air pollution on the nasal mucosa and nasal polyps of patients with CRS.

Recently, at the molecular level, the expression of oxidative stress- and inflammation-related genes in nasal polyps from patients with CRSwNP was evaluated. A significantly lower difference in the expression levels of transcription of antioxidant enzymes, including superoxide dismutase (SOD) and peroxiredoxin-2 (PRDX2), was reported, independent of age, sex, and smoking in patients with CRSwNP [12]. These results correspond to reduced SOD capacity with the increase in oxidative stress. Additionally, an analysis of the advanced oxidation protein products (AOPP) and SOD showed the opposite effect in patients with NP. The level of AOPP from NP was higher than in the healthy control group. However, SOD activity was lower, indicating that oxidative stress plays a vital role in the development of nasal polyps [13]. Another mechanism of regulating oxidative stress is mediated by the thioredoxin-interacting protein (TXNIP), which acts as a pro-oxidant protein by suppressing the activity of thioredoxin (TRX) and its antioxidant function [14]. However, in nasal tissue samples from patients with CRSwNP, the protein and mRNA of TXNIP and TRX were significantly increased and decreased, respectively, compared with the control subjects [15].

The transcription factors (TFs) are the primary regulators of gene expression. In this sense, essential TFs are related to oxidative stress, such as nuclear erythroid 2-related factor 2 (Nrf2), which regulates several antioxidant genes. For example, Nrf2 was necessary for an antioxidant pathway in a mouse model of rhinosinusitis. Knockout mice showed enhanced severity of eosinophilic sinonasal inflammation from disruption of the epithelial-specific Nrf2 pathway [16] and enhanced susceptibility to eosinophilic sinonasal inflammation [17]. This transcription factor has also been related to the stability of the sinonasal epithelial cell barrier function [18]. Scavenger receptors (SRs) are a broad family of transmembrane receptors involved in a dysfunctional host–environment interaction through a reaction with ROS production. Lectin-like oxidized LDL receptor-1 (LOX-1) is one member of these transmembrane receptors. In 2020, Nishida and colleagues found a significant increase in the mRNA expression levels of LOX-1 in CRSwNP patients [19]. 

Moreover, human sinuses are the primary source of NO in the airways. NO plays a role in regulating airway inflammation through the expression of NO synthase isoforms. Oxidative damage to the cellular components occurs when excessive amounts of NO are produced. Therefore, measuring NO levels can help diagnose CRS and sinonasal inflammation [20]. Additionally, dupilumab, an anti-IL-4 receptor alpha monoclonal antibody, has been used recently in the treatment of CRSwNP. Patients with CRSwNP treated with dupilumab were evaluated through extended nitric oxide analyses (exhaled, FENO; bronchial, JawNO; alveolar, CalvNO components; nasal, nNO) where the results showed that nitric oxide significantly improved after 15 days of treatment [21].

Some patients with CRSwNP suffer from bacterial airway infection and damage to the respiratory epithelia. TAS2R38 is an essential receptor in epithelial cells; its stimulation increases the production of NO, then the NO damages bacterial membranes, enzymes, and DNA, and increases the ciliary beat frequency. The expression of TAS2R38 in the cilia of human sinonasal epithelial cells is associated with susceptibility to CRS. Patients with advanced CRSwNP showed reduced TAS2R38 receptor expression in the sinonasal mucosa [22]. Similar results were found in Italian patients with CRSwNP [23]. Another critical receptor in the epithelial cells of the human airway is the bitter taste receptor (T2Rs). T2Rs can stimulate endothelial NO synthase (eNOS), whereby NO enhances mucociliary clearance with antibacterial effects on ciliated epithelial cells [24]. This activation of T2Rs is associated with CRSwNP status and has been proposed as a biomarker [25]. Oxidation can activate the calcium-activated kinase (CaMKII); the role of this kinase has been reported in several models of asthma, CRSwNP, cardiovascular disease, diabetes mellitus, and cancer [26]. The expression of ox-CaMKII was measured in CRSwNP with other proteins such as indoleamine 2,3-dioxygenase 1, tryptophan 2,3-dioxygenase, and kynurenine. Oxidized CaMKII was increased in eosinophilic polyps [27]. 

Nasal polyps (NP) are a common inflammatory mass affecting from 0.2% to 5.6% of the population [28]. The etiology of NP is unclear because the factors involved in its occurrence include the genetic background, the immune system, anatomical differences, and environmental conditions. However, NP are associated with other chronic inflammatory respiratory diseases such as cystic fibrosis, AERD, respiratory allergies, and, as mentioned above, CRSwNP [29]. Epidemiologically, there is an association between air pollution and the increased prevalence of these respiratory diseases [30]. Exposure to air pollutants enhances the symptoms’ severity, resulting in an imbalanced concentration of free radicals and ROS such as NO^•^, HO^•^, O_2_^•−^, and H_2_O_2_. In this regard, some studies have explored the association of the development and pathogenesis of NP with oxidative stress and air pollution, although information is limited.

Since the nasal epithelium is the first barrier of entry for inhaled particles such as pollutants, it plays a crucial role in the formation of NP. Oxidative stress damages the epithelium and causes mucosal edema due to impaired ion transport. The intracellular Na^+^ increases, the Ca^2+^ moves into the cell, and intracellular K^+^ decreases [31]. Moreover, chronic exposure to air contaminants affects the concentration of H_2_O_2_ and IL-8 in the nasal epithelium, a physiological defense mechanism [32]. The increase in the cells’ permeability and the migration of inflammatory cells of the proliferative and secretory response is yet, another innate immune response mechanism. The release of cytokines by effector cells, and the activity of cyclooxygenase and lipoxygenase are also associated with the pathophysiology of NP [33].

One of the most critical lines of defense against ROS are enzymes crucial for the activity of antioxidant, such as SOD, catalase, glutathione peroxidase, and thiol reductase [13]. The expression and activity of SOD, which catalyzes the dismutation of superoxide anions, is lower in NP than in healthy mucosa, which is correlated with lower antioxidant blood levels in NP patients [34,35,36]. Different molecules are related to oxidative stress, for example, malondialdehyde (MDA) and free radicals are the products of lipid peroxidation of polyunsaturated fatty acids in cell membranes. These oxidant products have higher NP levels than control tissues [37,38]. Another compound is nitric oxide, which is released in response to inflammation. Nitric oxide is involved in antiviral and bactericidal activity but inhibits cell proliferation, DNA synthesis, and collagen production. In NP, NO^•^ reacts with oxygen, producing peroxynitrite, which is associated with progressive epithelial injury. In patients with nasal polyposis, there is a lower concentration of NO^•^ compared with healthy patients, which is related to the downregulation of the nitric oxide metabolism, in which dismutase is crucial for the modulation of its activity [37]. 

In the same way, SOD activity was decreased, and MDA increased in NP samples, as mentioned above. Another approach to studying the role of oxidative stress in NP is to examine how the apoptotic pathway is related. In 2021, Simsek and colleagues reported deficient apoptosis through the MAPK/JNK pathway in NP tissues, which may have a role in the pathogenesis and is consistent with previous reports [39].

To date, oxidative stress is increased in patients with CRSwNP. However, this condition has a multifactorial etiology, and the role of air pollution is unclear. However, airborne pollutants may contribute to the pathogenesis of these diseases through the expression of several transcription factors and receptors in sinonasal epithelial cells. Some of them have been proposed as biomarkers.

## 3. Asthma

Asthma is a complex condition that is heterogeneous and is characterized by the critical role of chronic airway inflammation and oxidative stress. The eosinophils, lymphocytes, neutrophils, and mast cells generate inflammatory mediators and ROS/RNS that negatively affect the redox balance [40,41]. Furthermore, these are the basis for identifying the actual Type 2 high and Type 2 low phenotypes [42]. In Type 2 asthma patients, environmental factors favor the release of alarmins from the respiratory epithelium, which induces the differentiation of naïve T cells into Th2 cells. Damaged cells release interleukins such as IL−6, IL-1β, nitric oxide (NO^•^), prostaglandin E2 (PGE2), and tumor necrosis factor α (TNFα); the principal marker in these patients is the sputum eosinophilia [43]. T2-low asthma patients are characterized by sputum neutrophilia secondary to the activation of the NLRP3 inflammasome and elevated IL-1β; the activation of Th1 and/or Th17 cells associated with the imbalance of Th17/Treg cells seems to play an essential role in the pathology of asthma [44]. The response to the combination of Th1, Th2, and Th17 and genetic predisposition induce permanent structural changes in T2-high and T2-low asthma patients [45,46]. The process of airway remodeling is driven by subepithelial fibrosis, thickening of the sub-basement membrane, increased airway smooth muscle mass, angiogenesis, and mucous gland hyperplasia [45]. 

An imbalance in the airway-reducing state is a determinant of the initiation and severity of asthma. The ability of an individual to ward off oxidative lung damage depends partly on their endogenous antioxidant systems and exogenous antioxidant intake [27]. Several groups have shown that the levels of enzymatic antioxidants such as SOD, catalase, and glutathione peroxidases, as well as heme oxygenase-1 (HO-1), thioredoxins, peroxiredoxins, and glutaredoxins, are decreased in the bronchoalveolar lavage, sputum, and serum of asthmatic patients [40,47,48].

Some factors increase the risk of the development of asthma. Among these, regular exposure through inhalation to oxidants derived from outdoor and indoor ambient air pollutants is on the list of factors that contributes to the progression of the disease [49,50,51]. Since the relationship between oxidative stress and the inflammatory response depends on each other, and genetic predisposition could modify their balance, there is interest in the role of the inflammatory process as an activator of oxidative stress [52]. Signaling pathways involving the inflammatory process and the oxidative response associated with the development of asthma are a current matter of evaluation. For example, the adenosine 5’ monophosphate-activated protein kinase (AMPK)/sirtuin 1 (Sirt1) and Nrf2/HO-1 pathway [53] and the nitrogen-activated protein kinase (MAPK) pathway that includes extracellular signal-regulated kinases (ERKs), c-Jun N-terminal kinase (JNK), and p38 [54] have been evaluated. Nrf2 potentiates the activity of the antioxidant response element (ARE) that synthesizes antioxidant proteins such as HO-1 [55]. Multiple phytochemicals involved in the immune response activate the Nrf2/HO-1 signaling axis. In this sense, the Nrf2/HO-1, NF-κB, and MAPK pathways are relevant therapeutic molecular targets in asthma [56,57,58,59,60]. 

The methodologies used to identify the molecular biomarkers associated with respiratory diseases are varied and range from the use of proteomics platforms to the use of real-time PCR. In 2021, Suzuki et al. evaluated the plasma proteome using an aptamer-base affinity proteomic platform (SOMAscan^®^) in 34 subjects with stable COPD and 51 subjects with asthma, detecting 1238 proteins within which stress markers were found, such as myeloperoxidase (MPO), heme oxygenase 2 (HMOX2), superoxide dismutase (Cu-Zn) (SOD1), peroxiredoxin-1 (PRDX1), and glutathione-S-transferase P1 (GSTP1) [61].

However, some markers are associated with oxidative stress-related cell damage, such as MDA, which can be measured by colorimetric techniques, high-performance liquid chromatography (HPLC), or LC/atmospheric pressure chemical ionization tandem mass spectrometry (LC/APCI–MS/MS). In asthmatic patients, the sputum measurement of MDA in the sputum discriminated between patients and controls with greater accuracy than the levels found in plasma, where it might be more difficult to evidence the redox imbalance due to comorbidities and lifestyle risk factors. In addition, 8-isoprostane and the oxidative DNA damage marker 8-oxo-7,8-dihydro-29-deoxyguanosine (8-OHdG) were also increased in the sputum from asthmatic patients compared with nonasthmatic controls in several studies [62].

The mitochondria are the organelles that contributes the most to the generation of reactive oxygen species, and its contribution to oxidative stress in asthma has also been evaluated. The mitochondria are also susceptible to oxidative stress; under such conditions, they undergo an adaptive response through mitochondrial biogenesis. In 2021, Carpagnano et al. determined that the mitochondrial DNA/nuclear DNA (mtDNA/nDNA) ratio was a marker of mitochondrial oxidative stress in the exhaled breath condensate (EBC) of 53 patients with severe asthma, 11 patients with mild to moderate asthma, and 12 healthy subjects. They found higher levels of exhaled mtDNA/nDNA in severe asthmatic patients compared with the mild-moderate and healthy controls; this may be useful for differentiating the asthma phenotypes [63]. 

It is crucial to take into account that the presence of oxidative stress is a factor that triggers asthma symptoms and contributes to the severity of the disease. Moreover, oxidative stress promotes corticosteroid insensitivity by disrupting glucocorticoid receptor (GR) signaling, leading to the sustained activation of proinflammatory pathways in immune cells and the airway’s structural cells [64,65]. 

As already described in this section, many methodological strategies and various target molecules are related to oxidative stress. However, specific biomarkers with clinical applications in asthma have not yet been found.

## 4. Chronic Obstructive Pulmonary Disease (COPD)

Chronic obstructive pulmonary disease (COPD) is a progressive disease characterized by an airflow limitation that is not fully reversible even with treatment [66]. Risk factors associated with the development of COPD are exposure to inhaling noxious particles, mainly tobacco and biomass smoke and outdoor pollutants [67]. Airborne particulate matter, ozone, and sulfur dioxide are related to an increased risk of exacerbation and mortality in those patients [68]. In the alveolar space, air pollutants prime the alveolar macrophages and neutrophils that, through pro-inflammatory transcription factors such as nuclear factor-κB (NF-κB) and mitogen-activated protein kinases (MAPKs), produce cytokines and chemokines. Likewise, these cells are a source of reactive oxygen and nitrogen species (ROS and RNS); the increased production of ROS/RNS affects phagocytosis and activates NF-κB and p38 MAPK, which enhance the expression of pro-inflammatory genes [69]. Oxidative stress plays a crucial role in the pathogenesis of COPD; the imbalance between the production of oxidants and antioxidant defenses may also contribute to the worsening of the disease during acute exacerbation [70]. Studies have measured hydrogen peroxide (H_2_O_2_) in the exhaled air condensate. They have found that this reactive species is increases even more during the exacerbation of the disease, which worsens the inflammatory response in COPD patients [71].

Exposure to nitrogen dioxide (NO_2_) and particulate matter (PM_10_ and PM_2.5_) impairs lung function. Dorion et al. studied 303,887 individuals aged 40–69 years and found that higher concentrations of PM_2.5_ (OR: 1.52, 95% CI: 1.42–1.62, per 5 µg·m^−3^), PM_10_ (OR: 1.08, 95% CI: 1.00–1.16, per 5 µg·m^−3^), and NO_2_ (OR: 1.12, 95% CI: 1.10–1.14, per 10 µg·m^−3^) were associated with an increase in the prevalence of COPD [72]. Serum biomarkers of oxidative stress are related to COPD severity; for example, increased plasma MDA, a product derived from polyunsaturated fatty acid oxidation, is associated with the impaired recovery after incremental exercise observed in COPD patients [73]. In an experimental model of COPD, Sokar et al. showed that rats treated with a combination of dexamethasone (Dex) and losartan (Los) demonstrated inhibited disease progression, and the MDA levels significantly decreased by 50.75% and the SOD levels increased by 45.22% [74].

As we indicated before, airborne particulate matter increases the risk of COPD. Exposure to PM_2.5_ impairs mucociliary clearance. Chronic exposure to cigarette smoke can induce cell death by activating the receptor interacting protein (RIP) kinases 1 and 3 that initiate the stimuli of necroptosis stimuli associated with inflammation, airway remodeling, and emphysema [75]. The antioxidant defense system includes nonenzymatic and enzymatic molecules that prevent the uncontrolled increase in ROS/RNS and neutralizes the oxidants’ adverse effects [76,77]. García-Valero et al. found the decreased expression of extracellular SOD in the alveolar, bronchial, and arteriolar walls of COPD patients compared with the control group (0.59 ± 0.64 vs. 1.39 ± 0.63, respectively; *p* < 0.05). Moreover, MDA was a better marker for identifying COPD patients [78]. SOD activity has also been used to evaluate the functional exercise capacity in COPD patients through the six-minute walking test (6MWT). In this study, SOD was an independent predictor of the functional capacity in COPD patients; its activity explained a significant percentage of the variability in 6MWT-derived outcomes such as the 6 min walking distance (6MWD) (23%) and the 6 min walking work (6MWW) (27%) [79]. 

Furthermore, the genetic variants of SOD1 (rs2234694) in COPD patients were associated with the risk and severity of COPD (OR = 0.15, *p* = 0.04). Interestingly, patients with the +35AC genotype also had a statistically significant increase in glutathione plasma levels and a lower level of carbonyls (*p* = 0.03, *p* = 0.04, respectively) compared with the control group [80]. These findings emphasize the role of antioxidant enzymes and the impact of their genetic variants in oxidative biomolecular damage and the progression of COPD.

Glutathione is one of the primary antioxidant defenses of the respiratory system, and enzymes participating in its biosynthesis are affected in COPD patients. For instance, the activity of glutathione peroxidase (GPx) in the whole blood or red blood cells of COPD patients was lower than in controls [81]. In contrast, studies assessing serum/plasma GPx activity did not show a statistical significance between COPD patients and the control group. These contrasting results suggest further impairment of the antioxidant defense mechanisms in COPD [82].

Gamma-glutamyltransferase (GGT) has been considered a new marker of oxidative stress. Sun et al. showed the increased activity of serum GGT in patients with acute COPD exacerbation compared with stable COPD patients and control subjects. The authors suggested that a level of 21.2 IU/L GGT could be associated with a diagnosis of COPD; meanwhile, 26.5 IU/L could predict the exacerbation of COPD [83]. 

Damage to several biomolecules occurs during the process of oxidative stress, and lipids are one of the first to be damaged. Paraoxonase 1 (PON1) has an essential role in preventing lipid damage. Current results regarding the participation of PON1 in the pathogenesis of COPD are inconclusive. A report about the activity and phenotype distribution in COPD patients and healthy individuals showed that COPD patients exhibited higher PON1 activity than the control group (199.1 vs. 129.2, *p* = 0.002) [84]. Several studies have shown that COPD has extrapulmonary consequences, with an impact on functionality and quality of life; these include a reduction in muscle mass and muscle weakness which are proportional to the severity of COPD and antioxidant capacity [85]. In this context, various thiol-based antioxidants can increase the thiol content in the lungs and, in association with nitric oxide (NO^•^), can produce stable S-nitrothiols (RS–NOs) [86]. There is a need for further research into antioxidant therapy for better control of COPD [87].

## 5. Idiopathic Pulmonary Fibrosis

Idiopathic pulmonary fibrosis (IPF) is a chronic, progressive, fibrosing interstitial pneumonia of unknown cause that is characterized by abnormal epithelialization, excessive tissue remodeling, and advanced fibrosis within the alveolar wall. The most substantial factor is aging [88]. However, alterations in the production and clearance of mucus, architectural distortion, and increased cough reflex sensitivity intervene in the development of IPF, suggesting a role for targeted therapies and multidisciplinary treatment [89]. IPF is characterized by the irreversible scarring of the distal lungs due to the excessive accumulation of the extracellular matrix (ECM), rendering the lung stiff and compromising its normal gas exchange function [90]. In addition, IPF is associated with uncontrolled fibroproliferation and the activation of alveolar epithelial cells (AECs), inflammation, and oxidative stress [91]. Alongside the fibrotic process and the proliferation of many cell types, oxidative stress plays an essential role in the development and progression of IPF [92,93]. For instance, inhibited NADPH oxidase activation in the macrophages and several profibrotic mediators may explain the decreased in vivo oxidative stress and the preservation of lung function in patients [94]. Another recent study showed an elevation in a mitochondrial anion carrier protein, uncoupling protein-2 (UCP2). UCP2 is highly expressed in human IPF lung myofibroblasts and aged fibroblasts [95]. Oxidative stress arises from an imbalance between ROS and RNS, which leads to cellular dysfunction and tissue damage but directly damages the lungs’ epithelium, favoring the development of fibrosis [96]. During fibrosis, the expression of thromboxane-prostanoid receptor (TBXA2R) was upregulated in fibroblasts in the lungs of patients with IPF. TBXA2R links oxidative stress to fibroblast activation during lung fibrosis, and TBXA2R antagonists have been proposed for treating pulmonary fibrosis [97]. Growth factor β (TGF-β) is known to be modulated by ROS. TGF-β stimulates the proliferation of fibroblasts and their differentiation into myofibroblasts [98]. Pyruvate kinase M2 (PKM2) promoted the progression of fibrosis by directly interacting with Smad7 and reinforcing transforming growth factor-beta1 (TGF- β1) signaling [99]. Oxidants may alter the nature of the surrounding ECM. In the lungs, alveolar inflammatory cells, including lymphocytes, macrophages, and neutrophils, produce ROS/RNS. In IPF patients, these inflammatory cells produce high levels of ROS/RNS in response to cytokines and growth factors and are involved in the underlying mechanisms. Given the critical role of oxidative stress in IPF, using antioxidant drugs may improve some aspects of the disease, such as 3’5-dimaleamylbenzoic acid (3’5-DMBA), which has demonstrated pro-apoptotic, anti-inflammatory, and anti-cancer properties and has been used in IPF treatment. 3’5-DMBA significantly reduced the expression of the genes involved in fibrogenesis. In addition, 3’5-DMBA lowered the GSH/GSSG ratio without promoting lipid oxidation [99].

## 6. Lung Cancer

**Figure 2:**
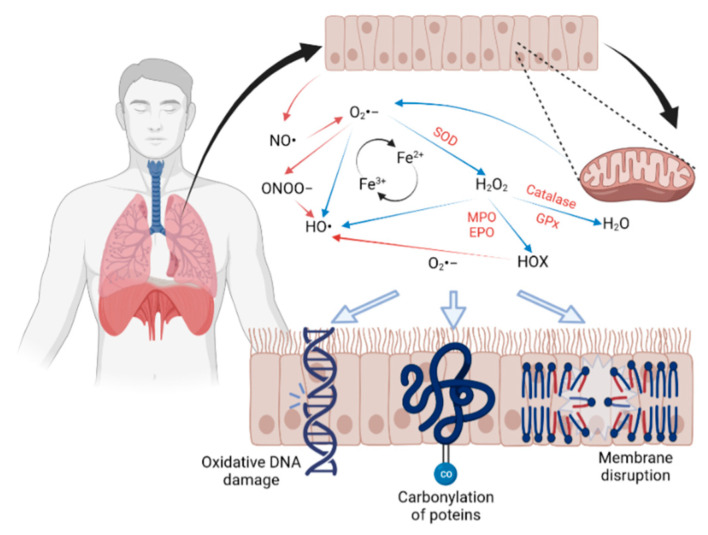
Air contaminants enter the respiratory tract. Once in contact with the epithelium, they increase the reactive oxygen and nitrogen species, which initiate a cascade of redox reactions that ultimately damage lipids, proteins, and DNA.

Oxidative stress can target biomolecules such as lipids, proteins, and even DNA/RNA, altering their structure and disrupting cellular functions (Figure 2). Lung cancer is associated with several risk factors, such as genetics and environmental exposures to xenobiotics such as air pollution and nutrition [100]. The International Agency for Research on Cancer (IARC) estimates that 1 out of 5 people will develop cancer once in their lives, and 1 out of 8 men and 1 out of 11 women will die as a consequence of the disease [101]. The global burden of cancer has increased to 19.3 million new cases, contributing to 10 million deaths during 2020, with lung cancer being the second most common type. Epidemiological data estimate that 2.2 million new cases and 1.8 million deaths represent 1 in 10 diagnoses (11.4%) and 1 in 5 deaths (18%) [102].

The evaluation of oxidative stress derived from air contaminants and their participation in the pathogenesis of cancer is complex because there is no exact amount of ROS/RNS related to cancer development. The regulation of several pathways by ROS/RNS, especially from the fine fraction of particulate matter (PM_2.5_), is also linked to its pathophysiology. Some of the organelles activated by noxious gases and particles are involved in redox pathways and are also associated with the development of cancer. The mitochondria and NADPH oxidase are both implicated in the generation of ROS/RNS; mitogen-activated protein kinase, phosphoinositide 3-kinase, and protein tyrosine phosphatase all participate in signal transduction cascades of covalent modifications. Nrf2, NF-κB, hypoxia-inducible factor (HIF), tumor protein p53 (p53), and activator protein 1 (AP-1) participate in transcription factors [103]. Among the environmental factors implicated in the pathogenesis of lung cancer, cigarette smoking contributes to more than 85% of the cases. Cigarette smoke contains over 5000 chemicals and at least 100 toxicants linked to many serious diseases, including cancer [104]. The relative risk of lung cancer in smokers varies from 10- to 30-fold compared with non-smokers; the risk increases depending on the smoking index. Several studies and meta-analyses have pointed out the participation of air pollution in the physiopathology of cancer. The scientific community recognizes that for every 10 micrograms per cubic meter (µg/m^3^) of increased exposure to PM_2.5_, the risk of dying from lung cancer rises by 36%. In Asia, the exposure to biomass fuels showed an odds ratio of 4.93 (95% CI: 3.73–6.52) [105].

The mitochondria are the major contributors to ROS; oxidative phosphorylation produces almost 90% of ROS. It is well-known that cancer cells obtain their energy through the glycolysis cycle, which is enhanced in these cells. Oxidative phosphorylation increases the production of O_2_^•−^, which gives rise to H_2_O_2_ and HO^•^ [106]. Mitochondrial DNA (mtDNA) has a high risk of ROS damage due to its proximity to the electron transport chain. The oxidative damage of DNA leads to the formation of carbon-centered radicals. 

Moreover, H_2_O_2_ generates HO^•^ via the Fenton reaction. Radical HO^•^ is considered t be the most reactive among all the oxygen species, and it can directly damage DNA and cause mutations. MDA and 4-hydroxynonenal (4-HNE) are reactive aldehydes derived from lipid peroxidation that react with an amino group of proteins and DNA bases, giving rise to mutagenic lesions [107]. Ye et al. performed a Kyoto Encyclopedia of Genes and Genomes analysis and found that mutations of mitochondrial energy metabolism pathway-related genes are essential for lung cancer. For example, the differential expression and mutation of some proteins such as glyceraldehyde-3-phosphate dehydrogenase (GAPDH), acyl-CoA synthetase bubblegum family member 1 (ACSBG1), cytochrome P450 family 4 subfamily A member 11 (CYP4A11), acyl-CoA oxidase 3 (ACOX3), and pristanoyl were related to poor prognosis [108]. The increased expression of Bcl-2/adenovirus E1B 19kDa-interacting protein 3 (BNIP3), a stress sensor protein, is associated with autophagy, dissemination, and poor prognosis in the early stages of non-small-cell lung cancer (NSCLC). Additionally, the decreased expression of sirtuin 3 (SIRT3) in lung cancer tissues and serum samples could be a promising biomarker for diagnosis with a sensitivity of 86.4%, a specificity of 94%, and a cutoff value of 3.12 [109,110].

Mammalian nicotinamide adenine dinucleotide phosphate (NADPH) oxidases (NOX) have been implicated in the tumorigenesis of lung cancer. The ubiquitous isoform NOX4 produces H_2_O_2_ continuously to regulate the physiological redox homeostasis. A hypoxic environment activates NOX4, via mRNA transcription and initiating protein translation, increasing its presence in multiple cancers [111]. NOX4 activates metabolic pathways related to tumor development; it regulates the ROS production of cancer cells [112]. Furthermore, the ROS produced by NOX4 stabilizes Nrf2 by inhibiting proteasomal degradation. It could also enhance tumor-associated macrophage infiltration and the pro-tumor function in some types of lung cancer by increasing the production of cytokines [113].

Moreover, NOX4 contributes to the inactivation of protein tyrosine phosphatase receptor Type J (PTPRJ or DEP-1) and negatively regulates the transformation of primary cells. NOX4 is a mediator of TGFb-RHO-ROCK-stimulated c-Jun N-terminal kinase (JNK) activation, which increases the expression of myofibroblast differentiation-related genes implicated in cancer progression and survival [114]. The inhibition of NOX function or mRNA expression slows tumor growth and promotes cancer cell death, hindering lung cancer formation and invasion [111].

The Nrf2 signaling system is the master regulator of the redox response. It is expressed at low concentrations by KEAP1 in the cytoplasm of all cell types due to its continuous proteasomal degradation in nonstressed circumstances. During oxidative stress, Nrf2 escapes from KEAP1 degradation, producing an increase in nuclear Nrf2 that allows the activation of the expression of the cytoprotective genes [115]. There may be more than 1000 cytoprotective genes regulated by Nrf2; the majority are related to detoxifying enzymes associated with the redox metabolism; the maintenance of reduced glutathione is an example [116]. The results of a meta-analysis performed by Wang et al. (2020) evidenced that high expression levels of Nrf2 were predictive of a poor survival rate, with a hazard ratio of 1.86 (95% CI: 1.44–2.41, *p* < 0.001) and were also a potential indicator of NSCLC tumor s’ aggressiveness [117].

On the other hand, the results of an in vitro study of human lung cancer cells (A549/DDP, a cisplatin-resistant A549, and H838 cell lines) demonstrated that treatment with metformin combined with cisplatin produced the dephosphorylation of Nrf2, accelerating its proteasomal degradation and overcoming chemoresistance in NSCLC [118]. There is still controversy regarding role of oxidative stress in cancer cells and the tumor environment. The participation of ROS/RNS in the pathophysiology of cancer seems to be cell-type- and context-dependent. The exact point at which oxidative stress favors the development and spread of such cells has been challenging to determine. More studies are needed to elucidate these controversies. As Paracelsus said, the dose makes the poison.

## 7. Conclusions

It is well established that patients with chronic respiratory diseases are susceptible to the damaging effects of air pollutants, which induce oxidative stress pathways and transcription factors ranging from early protective adaptations to inflammation and cell damage. Indeed, the large surface area for gas exchange makes the respiratory system a target for redox reactions where the metabolites generated attack cellular components, including protein structures, lipids, and DNA sequences, causing an imbalance between oxidants and antioxidants (Figure 2). Even though scientific groups have reported evidence related to air pollution and oxidative damage, there is still a long way to go. Studies are underway to evaluate the modulation of the redox pathway by PM_2.5_ in human-derived respiratory cells and its association with cellular damage. Finally, many antioxidant drugs that accelerate the conversion and inactivation of free radicals have been proposed as a treatment. However, to date, there are no highly effective therapies in the clinic, and further research is needed.

## Data Availability

Not applicable.
